# A rigid barrier between the heart and sternum protects the heart and lungs against rupture during negative pressure wound therapy

**DOI:** 10.1186/1749-8090-6-90

**Published:** 2011-07-08

**Authors:** Sandra Lindstedt, Richard Ingemansson, Malin Malmsjö

**Affiliations:** 1 Department of Cardiothoracic Surgery, Lund University and Skåne University Hospital, Lund, Sweden

## Abstract

**Objectives:**

Right ventricular heart rupture is a devastating complication associated with negative pressure wound therapy (NPWT) in cardiac surgery. The use of a rigid barrier has been suggested to offer protection against this lethal complication, by preventing the heart from being drawn up and damaged by the sharp edges of the sternum. The aim of the present study was to investigate whether a rigid barrier protects the heart and lungs against injury during NPWT.

**Methods:**

Sixteen pigs underwent median sternotomy followed by NPWT at -120 mmHg for 24 hours, in the absence (eight pigs) or presence (eight pigs) of a rigid plastic disc between the heart and the sternal edges. The macroscopic appearance of the heart and lungs was inspected after 12 and 24 hours of NPWT.

**Results:**

After 24 hours of NPWT at -120 mmHg the area of epicardial petechial bleeding was 11.90 ± 1.10 cm^2 ^when no protective disc was used, and 1.15 ± 0.19 cm^2 ^when using the disc (p < 0.001). Heart rupture was observed in three of the eight animals treated with NPWT without the disc. Lung rupture was observed in two of the animals, and lung contusion and emphysema were seen in all animals treated with NPWT without the rigid disc. No injury to the heart or lungs was observed in the group of animals treated with NPWT using the rigid disc.

**Conclusion:**

Inserting a rigid barrier between the heart and the sternum edges offers protection against heart rupture and lung injury during NPWT.

## Introduction

Cardiac surgery is complicated by poststernotomy mediastinitis in 1 to 5% of all procedures [1], and is a life-threatening complication [2]. The reported early mortality using conventional therapy is between 8 and 25% [3,4]. In 1999, Obdeijn and colleagues described the treatment of poststernotomy mediastinitis using vacuum-assisted closure [5], now called negative pressure wound therapy (NPWT). The technique entails the application of negative pressure to a sealed wound. NPWT has remarkable effects on the healing of poststernotomy mediastinitis, and has reduced the rate of mortality considerably [6].

There are, however, increasing numbers of reports of deaths and serious complications associated with the use of NPWT, where right ventricle rupture and bypass graft rupture resulting in death are the most devastating complications; the incidence being 4 to 7% of the patients treated for deep sternal wound infection with NPWT after cardiac surgery [7-9]. We have previously described the cause of heart rupture in pigs using magnetic resonance imaging [10,11]. The heart was shown to be drawn up towards the thoracic wall, the right ventricle bulged into the space between the sternal edges, and the sharp edges of the sternum protruded into the anterior surface of the heart [11]. Placing multiple layers of paraffin gauze over the anterior portion of the heart did not prevent deformation of the heart. However, these events could be prevented by inserting a rigid plastic disc between the anterior part of the heart and the inside of the thoracic wall [11].

The present study was conducted to investigate whether a rigid disc offers protection against heart and lung injury during NPWT. Sixteen pigs underwent median sternotomy followed by NPWT at -120 mmHg for 24 hours, in the absence (eight pigs) or presence (eight pigs) of a rigid plastic disc between the heart and the sternal edges. In the present article we measure epicardial bleeding after NPWT of a sternotomy wound. Petechial refers to one of the three major classes of purpuric conditions. The most common cause of petechial is through physical trauma. In the present article we believe that the epicardial bleeding is caused by trauma from the NPWT. The macroscopic appearance of the heart and lungs was inspected and the area of epicardial petechial bleeding was measured after 12 and 24 hours of NPWT.

## Material and methods

### Animals

A porcine sternotomy wound model was used. Sixteen domestic landrace pigs with a mean body weight of 70 kg were fasted overnight with free access to water. The study was approved by the Ethics Committee for Animal Research, Lund University, Sweden. All animals received humane care in compliance with the European Convention on Animal Care.

### Anesthesia and surgery

Premedication was performed with an intramuscular injection of xylazine (Rompun^® ^vet. 20 mg/ml; Bayer AG, Leverkusen, Germany; 2 mg/kg) mixed with ketamine (Ketaminol^® ^vet. 100 mg/ml; Farmaceutici Gellini S.p.A., Aprilia, Italy; 20 mg/kg). Before surgery, a tracheotomy was performed and an endo-tracheal tube was inserted. Anesthesia was maintained with a continuous infusion of ketamine (Ketaminol^® ^vet. 50 mg/ml; 0.4-0.6 mg/kg/h). Complete neuromuscular blockade was achieved by continuous infusion of pancuronium bromide (Pavulon; N.V. Organon, Oss, the Netherlands; 0.3-0.5 mg/kg/h). Fluid loss was compensated for by continuous infusion of Ringer's acetate at a rate of 300 ml/kg/h. Mechanical ventilation was established with a Siemens-Elema ventilator (Servo Ventilator 300, Siemens, Solna, Sweden) in the volume-controlled mode (65% nitrous oxide, 35% oxygen). Ventilatory settings were identical for all animals (respiratory rate: 15 breaths/min; minute ventilation: 8 l/min). A positive end-expiratory pressure of 5 cmH_2_O was applied. A Foley catheter was inserted into the urinary bladder through a suprapubic cystostomy. Upon completion of the experiments, the animals were given a lethal dose (60 mmol) of intravenous potassium chloride.

### Wound preparation for NPWT

A midline sternotomy was performed. The pericardium and the left and right pleura were opened. The wound was treated with NPWT in the presence or absence of a rigid plastic disc inserted between the heart and the sternum. A polyurethane foam dressing with an open-pore structure was trimmed so as to be slightly larger than the wound. The first layer was placed between the sternal edges. A second layer of polyurethane foam dressing was placed between the soft tissue wound edges. The wound was sealed with a transparent adhesive drape and connected to a vacuum source set to deliver a continuous negative pressure -120 mmHg.

### Experimental procedure

**Figure 1:**
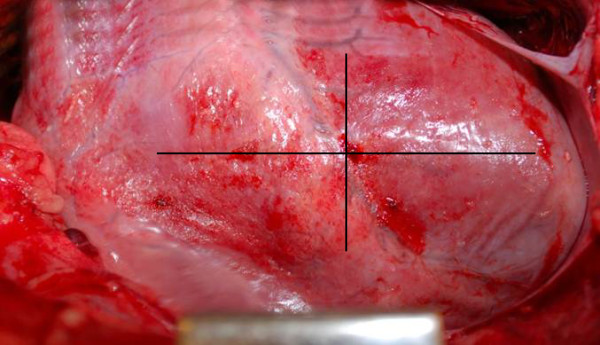
**Photograph of the heart after NPWT at -120 mmHg in the absence of a rigid barrier disc between the heart and the sternum**. It can be seen that the surface of the right ventricle of the heart is red and mottled due to epicardial petechial bleeding. The area of bleeding was determined by measuring the length and width.

The pigs were divided into two groups of eight animals. In one group a rigid barrier disc was inserted between the heart and the sternum before the application of NPWT, while the other group was exposed to NPWT without a disc. The animals were treated with a continuous negative pressure of -120 mmHg for 24 hours. The NPWT dressing was changed after 12 hours. The heart and lungs were inspected with regard to injury after 12 and 24 hours. The length and width of the area affected by petechial bleeding on the epicardial surface were measured and the area was calculated (Figure 1).

### Calculations and statistics

Calculations and statistical analysis were performed using GraphPad 5.0 software (San Diego, CA, USA). Statistical analysis was performed using the Mann-Whitney test when comparing two groups, and the Kruskal-Wallis test with Dunn's test for multiple comparisons when comparing three groups or more. Significance was defined as p < 0.05 (*), p < 0.01 (**), p < 0.001 (***), and p > 0.05 (not significant, n.s.). All differences referred to in the text have been statistically verified. Values are presented as means ± the standard error on the mean (S.E.M.).

## Results

### Heart injury

**Figure 2:**
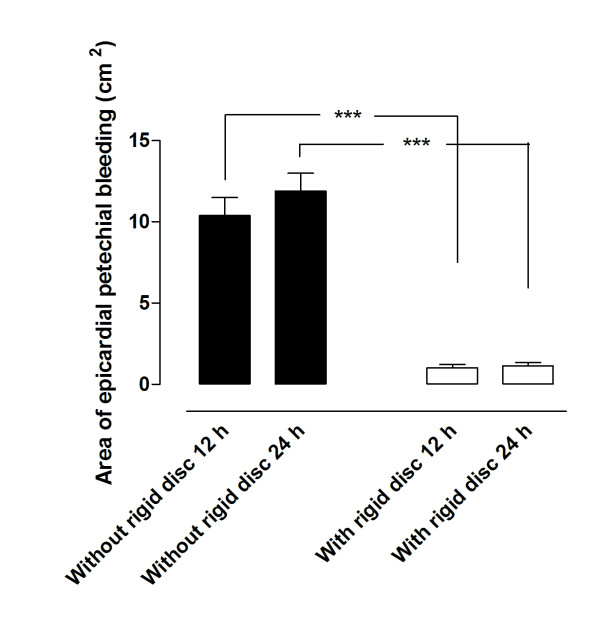
**Epicardial petechial bleeding following NPWT at -120 mmHg after 12 and 24 hours, with and without a rigid barrier disc between the heart and the sternum**. The area affected by petechial bleeding was measured. Results are presented as the mean of 8 values ± SEM. It can be seen that the area of epicardial bleeding was larger when NPWT had been performed without the rigid disc.

The surface of the right ventricle of the heart was red and mottled as a result of epicardial petechial bleeding in all cases following NPWT (Figure 1). After 12 hours of NPWT, the area of epicardial bleeding was significantly larger when NPWT had been performed without the rigid disc (10.40 ± 1.10 cm^2^) than with the disc (1.03 ± 0.20 cm^2^, p < 0.001, Figure 2). The area of epicardial petechial bleeding was only slightly larger after 24 hours of NPWT than after 12 hours (11.90 ± 1.10 cm^2 ^without the disc and 1.15 ± 0.19 cm^2 ^with the disc, Figure 2).

**Figure 3:**
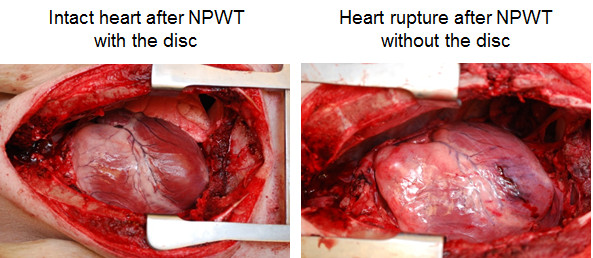
**Photograph of the heart in a porcine sternotomy wound treated with NPWT with (left) and without (right) a rigid disc between the heart and the sternum**. The right photograph shows heart rupture, which was seen in three of the eight pigs treated with NPWT without the disc. No heart rupture was seen in pigs treated with NPWT with the disc.

Right ventricular heart rupture was observed in three of the eight animals treated with NPWT without the rigid disc, while no ruptures were observed in the animals treated with NPWT when using the disc (Figure 3).

### Lung injury

**Figure 4:**
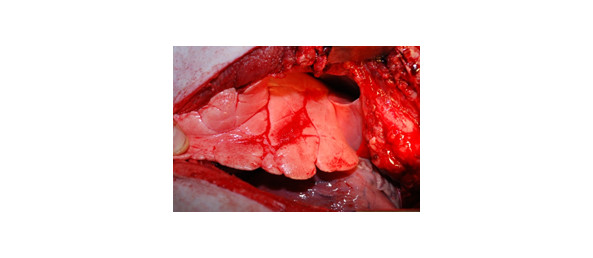
**Photograph of the right lung in a porcine sternotomy wound treated with NPWT without a rigid barrier disc between the heart and the sternum**. The photograph shows lung rupture, which was found in two of the eight pigs treated with NPWT without the disc. No lung ruptures were seen in pigs treated with NPWT with the disc.

Lung ruptures were observed in two of the eight animals treated with NPWT without the disc, while none was seen when using the disc (Figure 4). Lung contusion and emphysema were seen in all cases without the disc, while no such changes were observed when NPWT was applied with the disc.

## Discussion

The intention of this study was to investigate whether a rigid disc offers protection against heart and lung injury during NPWT. The results show both heart and lung injury after NPWT without the rigid disc. When NPWT is applied, the tissues are drawn together, towards the source of the vacuum. It is well known that NPWT results in wound contraction [12-16], however, the effect of the tissues deeper in the wound, such as the heart and lungs in the sternotomy wound, have been less well studied. In one of our previous studies using MRI it was shown that the heart and lungs were also drawn towards the vacuum [11]. This caused the right ventricle to bulge into the space between the sternal edges, and these sharp edges protruded into the anterior surface of the heart [11]. This is a plausible mechanism for the potentially hazardous events associated with NPWT.

The importance of protecting exposed organs during NPWT has received increasing attention recently, following reports of heart rupture and death. Sartipy *et al*. described 5 cases of injury following NPWT of deep sternal wound infection in Sweden, 3 of which were fatal [7]. In a series of 21 deep sternal wound infections treated with NPWT, Bapat *et al*. reported that one of a total of 5 mortalities was due to right ventricular rupture [17]. Ennker *et al*. also reported one fatality due to right ventricular rupture among 54 deep sternal wound infection patients treated with NPWT [8]. Khoynezhad *et al*. reviewed 3 of their own cases of heart rupture following NPWT, together with literature reports of 39 earlier cases, and found the incidence of heart rupture to be 7.5% [9]. They suggested that adhesion of the right ventricle to the infected sternal bone and soft tissues was a likely causative factor, but that the presence and mobility of the unstabilized sternal bone edge constituted a significant risk in the event of the patient breathing deeply or coughing [9].

In November 2009, the FDA filed an alert [18] to draw attention to this problem. We are now beginning to understand the mechanism underlying the injury to organs exposed to NPWT [11]. Inserting a rigid barrier between the heart and the sternum prevents the heart from being drawn up and deformed by the sternal edges [11]. To the best of the authors' knowledge, this study is the first study to test a protective device to prevent heart and lung injury during NPWT. In the present article we measure epicardial bleeding after NPWT of a sternotomy wound. Petechial refers to one of the three major classes of purpuric conditions. The most common cause of petechial is through physical trauma but might also be a sign of thrombocytopenia or as vasculitis. In the present article we believe that the epicardial bleeding (petechial seen on the surface of the epicardium) is caused by trauma from the NPWT. We believe that the NPWT in combination with sharp sternal edges are two most important factors resulting in complications as right ventricular heart rupture and bypass graft rupture, whereas the sharp sternal edges are the most important factor. Here we present evidence that inserting a rigid barrier between the heart and the sternum effectively prevents injury to the heart and lung during NPWT. A rigid barrier may thus be a clinically practicable device, offering protection to exposed organs in NPWT.

In summary, right ventricle rupture is a feared complication of NPWT in sternotomy wounds. The cause may be that the heart and lungs are drawn up towards the anterior thoracic wall and forced against the sharp sternal edges during NPWT. Inserting a rigid barrier between the heart and the sternum offers protection against heart rupture and lung injury during NPWT.

## Competing interests

The authors declare that they have no competing interests.

## Authors' contributions

SL, MM, and RI carried out the animal studies. SL, MM, and RI carried out the design of the study and MM and SL performed the statistical analysis. All authors read and approved the final manuscript.
